# Prediction of Postoperative Intravesical Recurrence Using Urine DNA Monitoring in Nonmuscular‐Invasive Urothelial Bladder Cancer

**DOI:** 10.1111/iju.70293

**Published:** 2025-12-09

**Authors:** Masashi Shiozaki, Tomonori Minagawa, Hitoshi Yokoyama, Yosuke Hirotsu, Toshio Oyama, Masao Omata, Yoshiyuki Akiyama

**Affiliations:** ^1^ Department of Urology Shinshu University School of Medicine Nagano Japan; ^2^ Department of Urology Yamanashi Prefectural Central Hospital Yamanashi Japan; ^3^ Genomic Analysis Center Yamanashi Prefectural Central Hospital Yamanashi Japan; ^4^ Department of Pathology Yamanashi Prefectural Central Hospital Yamanashi Japan; ^5^ Department of Gastroenterology Yamanashi Prefectural Central Hospital Yamanashi Japan; ^6^ The University of Tokyo Tokyo Japan

**Keywords:** intravesical recurrence, urine cytology, urine DNA, urothelial bladder cancer

## Abstract

**Objectives:**

Considering the high frequency of intravesical recurrence in bladder urothelial carcinoma (UBC), accurate non‐invasive biomarkers for recurrence prediction are needed for better management after transurethral surgery. This pilot study examined the usefulness of urine DNA for detecting and predicting intravesical recurrence in UBC.

**Methods:**

Patients with primary nonmuscle‐invasive UBC were prospectively enrolled just after initial transurethral surgery. Genomic profiles were evaluated in resected specimens of the original tumor and in urine during follow‐up. Urine DNA, urine cytology, and cystoscopy were all evaluated at 3‐month intervals until 1 year postoperatively. Upon centrifuging urine samples into precipitation and supernatant fractions, we retrospectively evaluated urine DNA using a genomic panel established in our previous report.

**Results:**

In the 19 patients enrolled, intravesical recurrent tumors were detected in six patients by cystoscopy. Urine DNA was positive in all of the recurrence cases before or at the time of cystoscopic or cytological detection, whereas urine cytology did not test positive before cystoscopic recurrence. Both urine precipitation and supernatant samples tested positive in 5 of 6 recurrence cases, indicating no obvious differences in the fraction used. Urine DNA was positive in 4 of the 13 nonrecurrence cases, among which three tested negative following Bacille Calmette‐Guerin therapy.

**Conclusions:**

Urine DNA as screened by our genomic panel may be useful for predicting and detecting intravesical recurrence in UBC. The simultaneous evaluation of urine precipitation and supernatant may enhance the clinical utility of urine DNA during surveillance for intravesical recurrence in UBC patients.

**Abbreviations**
BCG: Bacille Calmette‐Guerin
CNAs: copy number alterations
TURBT: transurethral resection of bladder tumor
UBC: urothelial bladder cancer

## 1 Introduction

Bladder cancer is one of the most common cancers, and more than 90% of bladder cancer is urothelial cancer, arising from the urothelium of the bladder. Most of the histological features about bladder cancer are reported as urothelial carcinoma (transitional cell carcinomas) [1]. Although roughly 75% of urothelial bladder cancer (UBC) cases are nonmuscle‐invasive, intravesical recurrence remains clinically problematic, especially in high‐risk UBC following transurethral resection of bladder tumor (TURBT) [2, 3]. The current standard of care for UBC diagnosis involves urine cytology and cystoscopy at 3‐ or 6‐month intervals after TURBT [1, 4]. Urine cytology has high specificity but low sensitivity, especially for low‐risk lesions for detection of UBC. In contrast, cystoscopy is highly invasive and has limited capability to indicate upper urinary tract recurrence and carcinoma in situ, although cystoscopy is a highly sensitive examination [5, 6]. Moreover, the results of these tests are observer‐dependent. On the other hand, the usefulness of urine DNA has been reported as a promising noninvasive biomarker for diagnosing UBC [7, 8, 9]. The detection of molecular residual disease and survival prediction after TURBT have also been highlighted for urine DNA [10]. In recent years, various urinary assays, especially epigenetic methods including DNA methylation analysis, have been used to detect UBC as promising biomarkers [11, 12]. On the other hand, we recently described the superiority of precipitation and supernatant urine DNA to diagnose UBC as compared with conventional urine cytology, showing a more precise reflection of tumor mutation profiles in a cross‐sectional study [13]. Accordingly, the present investigation hypothesized that assessing urine DNA could be beneficial in patients with UBC to monitor for intravesical recurrence after TURBT. This longitudinal pilot study sought to clarify the potential role of urine DNA in the detection and prediction of intravesical recurrence in UBC.

## 2 Methods

### 2.1 Study Design

This was a prospective longitudinal observational study. All patients were enrolled at Yamanashi Central Hospital (Yamanashi, Japan) after providing written informed consent to participate. The study was approved by the Ethics Committees of Yamanashi Central Hospital (no. G‐2018‐1).

### 2.2 Patient Eligibility

Eligible patients included those with pathologically confirmed primary nonmuscle‐invasive UBC of any age, sex, or performance status. The exclusion criteria were no informed consent, a history of surgery or radiation therapy for pelvic organ malignant disease, transurethral surgical management of benign prostate hyperplasia, refractory urinary tract infection, and urethral stricture. Bacille Calmette‐Guerin (BCG) intravesical therapy was recommended for all patients, apart from those with a single pTa UBC. Ultimately, BCG was independently performed from the protocol of this study according to the patient's decision.

### 2.3 Interventions and End‐Points (Protocol)

#### 2.3.1 Informed Consent, Patient Inclusion, and Baseline Evaluation

After the diagnosis of nonmuscle‐invasive UBC by TURBT, written informed consent was obtained from all enrolled participants for the analysis of age, gender, and genomic profiles of resected UBC. The 71 UBC genomic items in this study are described in the subsection “Gene selection, targeted sequencing, and data analysis.”

#### 2.3.2 Follow‐Up Strategy and Protocol

Both the precipitation and supernatant fractions of urine DNA were evaluated concurrently with urine cytology and cystoscopy, a total of five times at 3‐month intervals following TURBT. The results of urine cytology and urine DNA were then compared based on the event of recurrence as diagnosed by cystoscopy. The follow‐up period was set as 1 year after TURBT or the detection of intravesical recurrence.

#### 2.3.3 Tumor Sample Preparation and Histology

Tumor samples were obtained by TURBT just before recruitment in this study and fixed using 10% buffered formalin [14]. Laser capture microdissection was carried out to enrich tumor purity, as described previously [15]. Tumor DNA was extracted using the GeneRead DNA FFPE kit (Qiagen).

#### 2.3.4 Urine Sample Acquisition and Preparation

Urine precipitate (cellular fraction) and urine supernatant (noncellular fraction) were collected after centrifugation as reported previously [13]. The methodology of obtaining samples and detecting urine DNA was identical to that described in our previous report [13]. Briefly, DNA was extracted from urine precipitation and supernatant fractions with the QIAamp DNA Blood Mini QIAcube Kit (Qiagen, Hilden, Germany), and DNA concentration was determined using Nano Drop 2000 (Thermo Fisher Scientific, Waltham, MA, USA). DNA was extracted from urine supernatant samples with the MagMax Cell‐Free DNA extraction kit and the KingFisher Duo Prime (Thermo Fisher Scientific).

#### 2.3.5 Gene Selection, Targeted Sequencing, and Data Analysis

The genomic panel used in this study was originally developed at our institution. We analyzed Cancer Genome Atlas data and the literature [16, 17, 18, 19] to select 71 significantly mutated genes related to urological cancer (UBC, kidney cancer, and prostate cancer). A total of 3652 primer pairs were contained within the Urology Panel spanning 365.34 kb. Targeted sequencing of the 71 genes was carried out, including primary tumor and urine DNA (supernatant and precipitation) samples from the enrolled patients. These 71 genes were used in accordance with our previous reports for UBC detection [13, 19] and have been listed in Table 1. The library concentration was determined using an Ion Library Quantitation Kit. Emulsion polymerase chain reaction and chip loading were performed on an Ion Chef with an Ion PI Hi‐Q Chef kit. Sequencing was done with an Ion Proton Sequencer (Thermo Fisher Scientific). Sequence data analysis was carried out as described previously [12, 19]. Actionable mutations were referred to the OncoKB database (June 21, 2019 update) from the Memorial Sloan Kettering Cancer Center (https://www.oncokb.org) [20]. The genomic profiles of the recurrent cases were analyzed separately using TURBT specimens.

**TABLE 1:** Cancer comprehensive genomic panel using 71 genomic mutations.

AKT1	BRCA2	EGFR	GNAS	MDM2	NCOR2	PIK3R1	SPOP
APC	CCND1	EP300	HRAS	MED12	NF2	PTEN	STAG2
AR	CDK12	ERBB3	IDH1	MET	NFE2L2	RAD51B	TCEB1
ARID1A	CDKN1A	ERCC2	KDM5C	MLH1	NKX3‐1	RAD51C	TP53
ARID2	CDKN2A	FANCD2	KDM6A	MSH2	NRAS	RAD51D	TSC1
ATM	CHD1	FAT1	KEAP1	MTOR	PALB2	RB1	TSC2
BAP1	CHEK2	FBXW7	KMT2C	MYC	PBRM1	RSPO2	VHL
BRAF	CTNNB1	FGFR3	KMT2D	NCOA2	PIK3CA	SETD2	ZBTB16
BRCA1	CUL3	FOXA1	KRAS	NCOR1	PIK3CB	SMARCB1	

#### 2.3.6 Definition of Positive Urine DNA

We used the following filtering parameters for variant calling: minimum number of variant allele reads ≥ 5, coverage depth ≥ 10, and variant allele fraction ≥ 2%. Urine DNA was defined as positive when the above parameters were matched in either urine precipitation or supernatant samples, and mutations in urine DNA were confirmed to be tumor‐specific by the tumor sample. To avoid sequencing errors and distinguish somatic mutations from SNPs, blood (buffy coat) samples were also analyzed in this study. Germline SNPs were excluded by performing a tumor‐normal paired analysis. Moreover, copy number alterations (CNAs) were defined as CNA values of ≥ 7 and 0, respectively.

#### 2.3.7 Definition of Positive Urine Cytology

Formalin‐fixed paraffin‐embedded DNA quality was analyzed as described previously [21]. Histological and cytological examinations were conducted in a blinded manner by a pathologist (T.O.) and a cytologist (K.A.), respectively.

## 3 Results

### 3.1 Patient Characteristics

The clinical characteristics of the enrolled patients are shown in Table 2. We analyzed a total of 19 patients (all male) with UBC who received TURBT at a median age of 70.8 years. Intravesical recurrence was detected in six patients (31.6%) by cystoscopy in the 1‐year post‐TURBT observation period. The mean period for recurrence detection was 7 months after TURBT. Thirteen patients received BCG intravesical therapy, and urine samples were acquired during or after BCG intravesical therapy in all of the patients.

**TABLE 2:** Characteristics of enrolled patients.

Total		19
	Male	19
	Female	0
Age (mean years)		70.8
Presurgical cytology		
	Class I	3
	Class II	0
	Class III	4
	Class IV	1
	Class V	9
	N/A	1
T stage of primary tumor		
	Ta	13
	Tis	1
	T1	5
Cellular atypism of primary tumor		
	G2	17
	G3	2
Single or multiple tumor		
	Single tumor	9
	Multiple tumor	10
Treatment after TURBT		
	BCG	13
	None	6
Recurrence during protocol (within 1 year after TURBT)		
	Recurrence	6
	No recurrence	13

Abbreviations: BCG, Bacille Calmette‐Guerin; TURBT, transurethral resection of bladder tumor.

### 3.2 Genomic Profile of Resected Tumors

**FIGURE 1:**
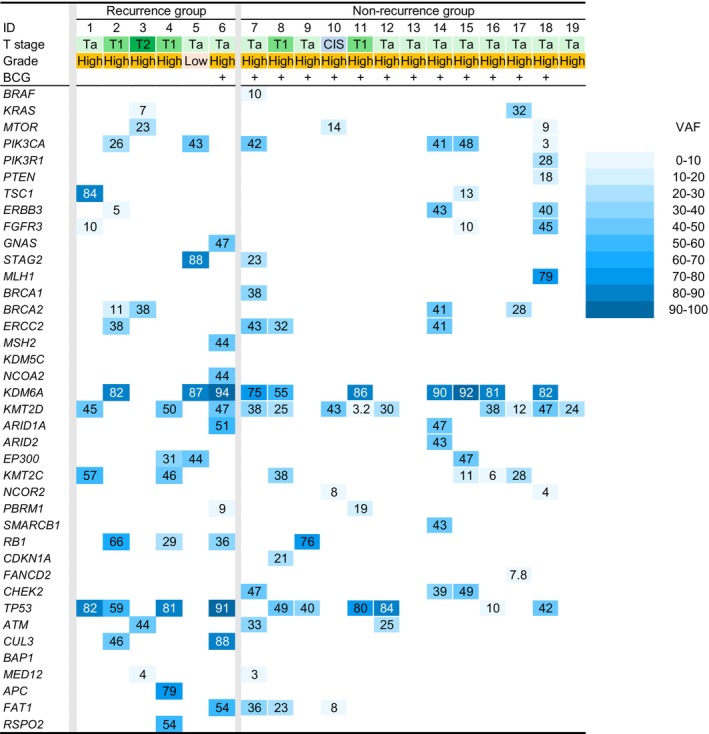
The genomic profiles and variant allele frequencies derived from TURBT specimens. Six cases of the Recurrence group and 13 cases of the Nonrecurrence group were demonstrated. Genomic mutation was not detected in only 1 tumor (Case 13). Abbreviations: BCG, Bacille Calmette‐Guerin; TURBT, transurethral resection of bladder tumor; N/A, not applicable.

At least 1 genomic mutation was detected in 18 of the 19 tumors. The genomic profiles and variant allele frequencies are presented for the 6 recurrence cases and 12 nonrecurrence cases in Figure 1. Notably, *KMT2D* was detected in 12 cases, while *KDM6A* and *TP53 w*ere identified in 10 cases each. The full information on the detected mutation regarding the resected tumor is noted in Table S1.

### 3.3 Urine DNA, Urine Cytology, and Cystoscopic Findings in Recurrence and Nonrecurrence Cases

The time‐course results for precipitate and supernatant urine DNA, urine cytology, and cystoscopy are listed for the recurrence and nonrecurrence groups in Table 3. Genomic positivity in the urine precipitate and supernatant was observed in all recurrence cases, whereas cytological positivity was detected in 2 of 6 (33%) cases (Cases 1 and 6) at the time of cytoscopic detection. Positive urine DNA in 5 cases was detected ahead of cystoscopic recurrence, apart from 1 case (Case 1) that was positive in all examinations during the initial follow‐up period. On the other hand, positive urine DNA was detected in the initial follow‐up period in 3 of the 13 nonrecurrence cases (Cases 7–9), which became negative during the follow‐up period. Positive urine DNA was detected from 6 to 12 months after TURBT in only 1 case (Case 11), and recurrence in the upper urinary tract was clinically ruled out using urine examination, urine cytology, and ultrasonography, computed tomography for more than 3‐year follow‐up after TURBT. The full information of the detected mutation about the urine samples (precipitation and supernatant fractions) is noted in Table S2. Summarized results about quality check of obtained samples are also supplementally presented in Table S3. Moreover, the results of urine examination are shown in Table S4 to exclude the influence of hematuria, pyuria, and so on.

**TABLE 3:** Time course and results of each examination during the study period.

Follow‐up period	Case no.	Grade	BCG	3 months after TURBT				6 months after TURBT				9 months after TURBT				12 months after TURBT			
Examination				U‐ppt	U‐sup	Cyto	Cyst	U‐ppt	U‐sup	Cyto	Cyst	U‐ppt	U‐sup	Cyto	Cyst	U‐ppt	U‐sup	Cyto	Cyst
Recurrence group	1	High	—	+	+	+	+												
	2	High	—	+	+	—	—	+	—	—	+								
	3	High	—	—	+	—	—	+	—	—	+								
	4	High	—	—	+	—	—	+	—	—	+								
	5	Low	—	—	+	—	—	—	—	—	—	—	—	—	+				
	6	High	+	—	—	—	—	—	—	—	—	—	+	—	—	—	—	+	+
Nonrecurrence group	7	High	+	+	+	—	—	—	—	—	—	—	—	—	—	—	—	—	—
	8	High	+	+	+	+	—	—	—	—	—	—	—	—	—	—	—	—	—
	9	High	+	+	—	+	—	—	—	—	—	—	—	—	—	—	—	—	—
	10	High	+	—	—	+	—	—	—	—	—	—	—	—	—	—	—	—	—
	11	High	+	—	—	—	—	—	—	—	—	—	—	—	—	—	+	—	—
	12	High	+	—	—	—	—	—	—	—	—	—	—	—	—	—	—	—	—
	13	High	+	—	—	—	—	—	—	—	—	—	—	—	—	—	—	—	—
	14	High	+	—	—	—	—	—	—	—	—	—	—	—	—	—	—	—	—
	15	High	+	—	—	—	—	—	—	—	—	—	—	—	—	—	—	—	—
	16	High	+	—	—	—	—	—	—	—	—	—	—	—	—	—	—	—	—
	17	High	+	—	—	—	—	—	—	—	—	—	—	—	—	—	—	—	—
	18	High	+	—	—	—	—	—	—	—	—	—	—	—	—	—	—	—	—
	19	High	—	—	—	—	—	—	—	—	—	—	—	—	—	—	—	—	—

*Note:* “+” indicates an intravesical BCG therapy or a positive examination result. “‐” indicates no intravesical BCG therapy or a negative examination result of each examination. Blank indicates an unadministered examination after diagnosis of bladder cancer recurrence on cystoscopy.

Abbreviations: BCG, Bacille Calmette‐Guerin; Cyst, cystoscopy; Cyto, cytology; TURBT, transurethral resection of bladder tumor; U‐ppt, urine precipitate; U‐sup, urine supernatant.

### 3.4 Genomic Profile of Recurrence Cases

The genomic profiles of the six recurrence cases positive for urine DNA are summarized in Table 4. The genomic mutations were mainly of tumor suppressor genes: *KMT2D, KDM6A*, and *TP53*, which were frequently positive in the original tumors, were detected in 2, 1, and 2 cases, respectively. All of the genomic mutations were also found in the resected tissue in each case. No CANs were found in the tumor tissue, and were not assessed for the urine samples.

**TABLE 4:** Genomic profiles and variant allele frequencies.

ID	Specimen	Locus	Gene	VAF
1	U‐ppt & U‐sup	chr4:1807841	*FGFR3*	5
	U‐ppt & U‐sup	chr7:151949114	*KMT2C*	21
	U‐ppt & U‐sup	chr9:135776990	*TSC1*	29
	U‐ppt & U‐sup	chr12:49416500	*KMT2D*	25
	U‐ppt & U‐sup	chr17:7576855	*TP53*	25
2	U‐ppt	chr2:225362567	*CUL3*	45
	U‐ppt & U‐sup	chr13:49027128	*RB1*	63
	U‐ppt & U‐sup	chr3:178936091	*PIK3CA*	41
	U‐ppt & U‐sup	chr17:7578239	*TP53*	49
	U‐ppt & U‐sup	chr19:45871992	*ERCC2*	36
	U‐ppt & U‐sup	chrX:44942718	*KDM6A*	78
3	U‐ppt & U‐sup	chr11:108143533	*ATM*	5
4	U‐ppt & U‐sup	chr5:112177105	*APC*	7
	U‐ppt & U‐sup	chr7:151841945	*KMT2C*	17
	U‐ppt & U‐sup	chr7:151846079	*KMT2C*	14
	U‐ppt & U‐sup	chr12:49438225	*KMT2D*	10
	U‐ppt & U‐sup	chr17:7579418	*TP53*	5
	U‐ppt	chr22:41573200	*EP300*	7
5	U‐sup	chr22:41562653	*EP300*	7
6	U‐sup	chr1:27106538	*ARID1A*	5.7
	U‐sup	chr20:57480474	*GNAS*	4.4

Abbreviations: U‐ppt, urine precipitate; U‐sup, urine supernatant; VAF, variant allele frequency.

## 4 Discussion

Previously, we identified 168 somatic mutations in primary UBC using samples from urine supernatant, urine precipitation, and plasma [13]. In the report, at least one mutation identical to mutations in the corresponding primary tumor was observed in 72% (18/25) of patients by genomic analysis of urine supernatant, 76% (19/25) by urine precipitation, and 8% (2/25) by plasma. In the current longitudinal study, the usefulness of positive urine DNA findings was confirmed in comparisons with cytological findings. Five positive cases of urine DNA were detected prior to positivity on cystoscopy, which suggested that urine DNA could be employed to predict cytoscopic recurrence after TURBT. In contrast, negative urine DNA findings may indicate a decreased risk of intravesical recurrence. Three positive cases of urine DNA in the nonrecurrence group (Cases 7–10) had become negative 6 months after TURBT. These findings might be reflected by clinically benign lesions with malignant potential and treatment effects of BCG therapy on clinically undetectable lesions. Only 1 case (Case 11) was positive for urine DNA 6–12 months post‐TURBT, which suggested a potential risk of recurrence after the study protocol. Although we performed this pilot investigation with a small cohort, the potential predictive ability for intravesical recurrence and estimation of treatment effects were evident from our results. The superior sensitivity and specificity of urine DNA may help support urine cytology findings.

Recently, genomic profiling from liquid biopsy has been used to diagnose malignant diseases and prescribe appropriate treatment. Although liquid biopsy is a promising option for the early detection of malignant diseases, obtaining DNA from blood samples remains problematic from the viewpoint of sensitivity, especially in low‐grade cancers. Indeed, intravesical recurrence represents an important clinical problem after TURBT even in low‐grade UBC. Refinements to liquid biopsy and other biomarkers are needed for follow‐up after TURBT. Tamura et al. reported on the usefulness of circulating tumor DNA in urine and blood for detecting recurrence after urothelial cancer in the upper urinary tract [22]. Elsewhere, Abe et al. employed urine pellet DNA to monitor for intravesical recurrence following TURBT in noninvasive UBC, suggesting the possibility to reduce cystoscopy frequency [23].

Urine DNA, especially cell‐free DNA, has emerged as a promising biomarker to detect several malignant diseases, including cancers of the kidney, lung, liver, and colon [24, 25, 26, 27, 28]. Cell‐free DNA can diagnose UBC, detect molecular residual UBC, and predict survival [6, 9]. Urine‐based tumor DNA may also serve as a tumor marker for screening, diagnosing, and monitoring UBC [29].

Despite numerous reports on the diagnosis of UBC, longitudinal investigations on urine DNA for UBC prediction during follow‐up after TURBT are scarce. Abe et al. described the clinical validity of urine pellet DNA to monitor UBC recurrence [23], which is nearly synonymous with the cell‐free DNA obtained from urine supernatant. On the other hand, both the urine precipitate and supernatant were investigated in our study. Although no remarkable differences between the fractions were detected, additional comparative studies are needed, as urine precipitate may be influenced by inflammation and infection, while malignant diseases in other organs besides the urinary tract may affect urine supernatant [7].

To the best of our knowledge, this study is the first to explore the relationship of urine DNA, both precipitate and supernatant, for monitoring the intravesical recurrence of UBC. However, several limitations should be considered when interpreting the results. First, the patient background including the number of enrolled patients and follow‐up period was too limited, mainly for economic reasons, to establish firm evidence on urine DNA. Moreover, the patient cohort was homogenous, consisting of all males. However, the obtained results may serve as a preliminary step in establishing the usefulness of urine DNA for UBC in the clinical setting. Further multicenter investigations should include larger diverse cohorts for longer follow‐up periods. Second, the genomic panel that was originally made and used in this study may also represent a limitation. The selected genomic mutations were theoretically and practically determined to detect UBC. However, future studies are needed to explore combinations of more optimal genomic mutations, such as *TERT* and *HRAS*. Third, all of the participants in this study were male. Additional research in a wider range of UBC patients, including those with carcinoma in situ, is needed to confirm our results. Fourth, the differences between urine precipitate and supernatant were not clearly evident in this study. Although we had hypothesized that urine precipitate, which included cellular components, was superior to supernatant, no such discrepancies were apparent. Further investigations are required on whether the simultaneous evaluation of urine precipitation and supernatant has clinical utility in the surveillance of intravesical recurrence in UBC.

In conclusion, our results implicate urine DNA as a promising biomarker to detect and predict intravesical recurrence in UBC by compensating for the low sensitivity of conventional cytological examinations. Future studies should aim to optimize the accuracy of urine DNA, clarify the respective roles of precipitate and supernatant urine, and evaluate long‐term outcomes.

## Author Contributions

**Masashi Shiozaki:** writing – original draft, conceptualization, writing – review and editing, visualization, methodology, data curation. **Tomonori Minagawa:** writing – review and editing. **Hitoshi Yokoyama:** data curation. **Yosuke Hirotsu:** conceptualization, data curation, methodology. **Toshio Oyama:** data curation. **Masao Omata:** supervision. **Yoshiyuki Akiyama:** supervision.

## Funding

This work was supported by a Grant‐in‐Aid for the Genome Research Project from Yamanashi Prefecture (to M.O. and Y.H.), the Japan Society for the Promotion of Science (JSPS) KAKENHI Early‐Career Scientists JP18K16292 (to Y.H.), a Grant‐in‐Aid for Scientific Research (B) 20H03668 and 23K27646 (to Y.H.), a Research Grant for Young Scholars (to Y.H.), the YASUDA Medical Foundation (to Y.H.), the Uehara Memorial Foundation (to Y.H.), and medical research grants from the Takeda Science Foundation (to Y.H.).

## Disclosure

Approval of the Research Protocol by an Institutional Reviewer Board: The protocol for this research project has been approved by the Institutional Review Board of Yamanashi Central Hospital (no. G‐2018‐1). This study conforms to the provisions outlined in the Declaration of Helsinki.

Registry and the Registration No. of the Study/Trial: UMIN000058060.

Animal Studies (If Not Applicable Please Write N/A.): Not applicable.

## Consent

Informed written consent was obtained from all patients.

## Conflicts of Interest

Tomonori Minagawa and Yoshiyuki Akiyama are the Editorial Board members of the International Journal of Urology and the co‐authors of this article. To minimize bias, they were excluded from all editorial decision‐making related to the acceptance of this article for publication.

## Supporting information


**TABLE S1:** Full information of the detected mutation about the resected tumor

**TABLE S2:** Full information of genomic profile about urine samples

**TABLE S3:** Results of quality check

**TABLE S4:** Results of urine examination
